# ERK Activation Modulates Cancer Stemness and Motility of a Novel Mouse Oral Squamous Cell Carcinoma Cell Line

**DOI:** 10.3390/cancers12010061

**Published:** 2019-12-24

**Authors:** Yu-Lin Chen, Ko-Jiunn Liu, Chuan-Wei Jang, Chia-Chun Hsu, Yi-Chen Yen, Yi-Ling Liu, Tsung-Hsien Chuang, Ssu-Han Wang, Yu-Ke Fu, Ching-Chuan Kuo, Ya-Wen Chen

**Affiliations:** 1 National Institute of Cancer Research, National Health Research Institutes, Miaoli 35053, Taiwan; 2 National Institute of Cancer Research, National Health Research Institutes, Tainan 70456, Taiwan; 3 Immunology Research Center, National Health Research Institutes, Miaoli 35053, Taiwan; 4 Institute of Biotechnology and Pharmaceutical Research, National Health Research Institutes, Miaoli 35053, Taiwan; 5 Ph.D. Program for Aging, Graduate Institute of Biomedical Sciences, China Medical University, Taichung 40402, Taiwan

**Keywords:** oral squamous cell carcinoma, syngeneic mouse cell line, migration, cancer stemness, ERK activation

## Abstract

We established the NHRI-HN1 cell line from a mouse tongue tumor induced by 4-nitroquinoline 1-oxide (4-NQO)/arecoline, with further selection for cell stemness via in vitro sphere culture, to evaluate potential immunotherapies for oral squamous cell carcinoma (OSCC) in East and Southeast Asia. In vivo and in vitro phenotypic characterization, including tumor growth, immune modulator administration, gene expression, morphology, migration, invasion, and sphere formation assays, were conducted. NHRI-HN1 cells are capable of generating orthotopic tumors in syngeneic mice. Interestingly, immune stimulation via CpG oligodeoxynucleotide (CpG-ODN) dramatically reduced the tumor growth in NHRI-HN1 cell-injected syngeneic mice. The pathways enriched in genes that were differentially expressed in NHRI-HN1 cells when compared to non-tumorigenic cells were similar to those that were identified when comparing human OSCC and non-tumorous tissues. NHRI-HN1 cells have characteristics of epithelial–mesenchymal transition (EMT), including enhanced migration and invasion. NHRI-HN1 cells showed aggressive cell growth and sphere formation. The blockage of extracellular signal-regulated kinase (ERK) activation suppressed cell migration and reduced stemness characteristics in NHRI-HN1 cells, similar to human OSCC cell lines. Our data suggest that NHRI-HN1 cells, showing tumorigenic characteristics of EMT, cancer stemness, and ERK activation, are sufficient in modeling human OSCC and also competent for use in investigating oral cancer immunotherapies.

## 1. Introduction

Oral squamous cell carcinoma (OSCC), which is a subset of head and neck squamous cell carcinoma (HNSCC), is one of the most common human malignancies [1], and its incidence has risen recently [2]. Patients with OSCC continue to have poor prognosis, with an estimated five-year overall disease-free survival rate of ~50%, despite significant advances in conventional and combination treatments [3]. Targeted cancer therapy might provide more effective treatment and it is less harmful to normal cells. Until recently, cetuximab was the only targeted therapy approved for HNSCC [4]. Recently, the FDA (Food and Drug Administration) approved the immune checkpoint programmed cell death protein 1 (PD-1) inhibitor pembrolizumab for the treatment of HNSCC, regardless of human papillomavirus expression [5]. The activation of immune response leads to better tumor control, suggesting a crucial role for immunosuppression in disease progression. However, the treatment might impact immune response and therapeutic outcome in unexpected ways. Therefore, it is important to fully characterize immunoresponse to novel OSCC therapies in vivo.

Mouse models can closely mimic human oral squamous epithelial carcinogenesis and they are instrumental in studying tumor initiation and development, as well as testing new therapeutic strategies [6]. Traditionally, patient-derived xenograft models using immunocompromised mice have been instrumental in the development of novel cancer treatments. However, these models are not suitable for investigating the complicated interplay of tumor cells and host immune factors in malignant transformation and metastatic progression. Several immunocompetent mouse oral cancer model systems have been developed, but each of them has different limitations and challenges [7]. Genetically engineered mouse models have long latencies, limited tumor mutational burden, and minimal genetic diversity [8]. Chemically induced tumor models require protracted multi-step carcinogenesis. In contrast, syngeneic models have short latencies, high reproducibility, and a higher throughput. For these advantages, they have been heavily used in cancer immunology research for decades [7]. In East and Southeast Asia, most OSCC patients have betel quid chewing and smoking habits [9]. Unfortunately, few available mouse cell lines demonstrate the characteristics of OSCC in this region [10]. It is imperative to establish a mouse cell line that models the OSCC that is prevalent among East and Southeast Asian patients.

Appropriate mouse OSCC cell lines mimic the characteristics of human OSCC cells [9]. The sustained activation of p53, mitogen-activated protein kinase (MAPK), WNT, and PI3K/AKT/mTOR pathways signal proliferation in OSCC cells [11,12], and epithelial-mesenchymal transition (EMT) is a very conserved, naturally occurring transdifferentiation process by which a polarized epithelial cell develops a mesenchymal phenotype. The EMT of cancer cells has been associated with increased tumor invasiveness, metastasis, recurrence, and poor clinical outcomes [13]. The culmination of EMT is marked by an up-regulation of transcriptional repressors of epithelial genes or activators of mesenchymal genes, such as Snail, Slug, Zeb1/2, and Twist. The repressors inhibit E-cadherin expression, which is central to maintaining the epithelial phenotype. Concomitantly, the expression of mesenchymal proteins, such as N-cadherin, vimentin, fibronectin, fibroblast-specific protein-1, and α-smooth muscle actin (α-SMA) is increased. The actin cytoskeleton, cell morphology, and cell behavior also change dramatically during EMT [14]. Among cancer cells, cancer stem cells (CSCs) are a highly tumorigenic subpopulation that can self-renew and differentiate into heterogeneous cell types. CSCs are thought to play a role in tumor recurrence and metastasis [15], and they are considered one of the critical factors that contribute to tumor heterogeneity, aggressiveness, and resistance to therapy [13]. An important characteristic of CSCs is expression of pluripotent transcription factors, such as Oct4 and Nanog, which are well known for their role in reprograming somatic cells into a stem cell-like state. There is evidence that upregulated stemness-associated transcription factors play a role in tumorigenic transformation, tumorigenicity, metastasis, and distant recurrence after chemotherapy or radiotherapy [16].

In this study, we established and characterized a novel murine OSCC cell line, NHRI-HN1. We also demonstrated that NHRI-HN1 is highly tumorigenic in vivo and it can produce syngeneic tumors by orthotopic injection into immunocompetent hosts. The stimulation of host immunity dramatically affected the tumorigenic process, illustrating the model’s advantage for studying tumor-host immune system interactions. NHRI-HN1 cells demonstrated similar gene expression and signaling pathway modulation as compared to human OSCC tissues. As in human OSCC cell lines, MAPK kinase inhibitors suppressed NHRI-HN1’s enhanced migration and cancer stemness, which suggests an essential role for extracellular signal-regulated kinase (ERK) activation in modulating cell motility and stemness in NHRI-HN1 cells and human oral cancer cells.

## 2. Results

### 2.1. Establishment of Two Mouse OSCC Cell Lines from Carcinogen-Induced OSCC Mice

**Figure 1:**
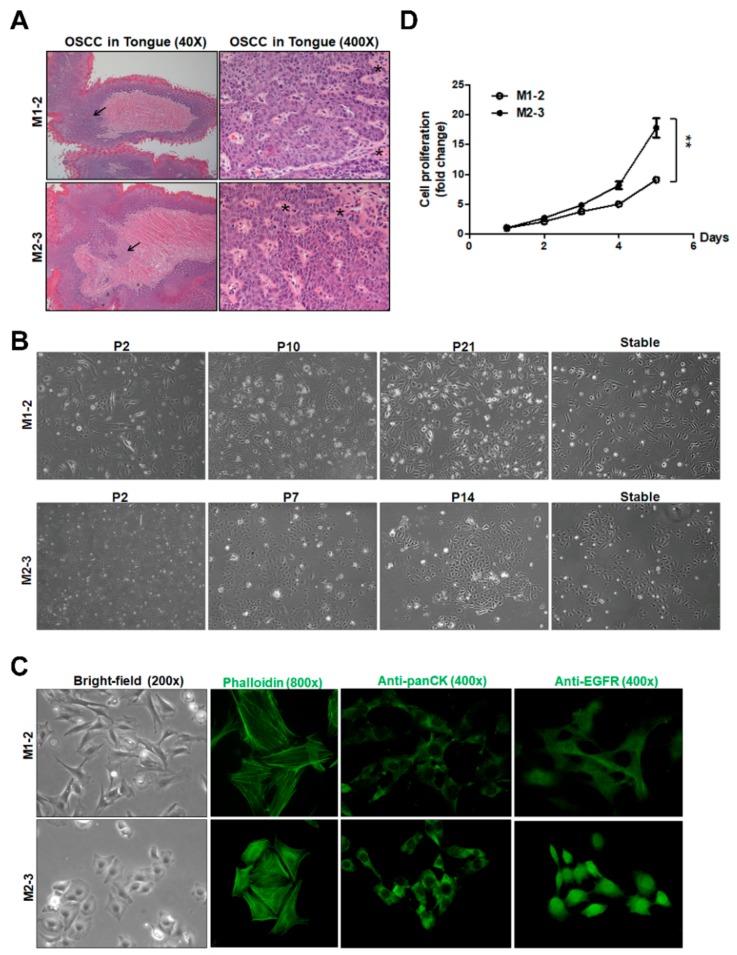
Establishment of two mouse oral squamous cell carcinoma (OSCC) cell lines from carcinogen-induced tongue tumors. (**A**) Histological examination of the carcinogen-induced tongue tumors at 40× (left panel) and 400× (right panel) magnifications. Black arrows indicate the tumor parts. Asterisks (*) indicate the representative keratinization and keratin-pearl formation. (**B**) Cell morphology of M1-2 and M2-3 cells at different passages at 100× magnification. (**C**) Representative images of cells at 200× magnification (left panel). Phalloidin (middle left panel), Pan-CK (middle right panel) and epidermal growth factor receptor (EGFR) staining (right panel) in M1-2 and M2-3 cells at 400× or 800× magnification. (**D**) A representative cell growth curve obtained by MTS assay in M1-2 and M2-3 cells. The results were expressed as the fold change to the results at day 1. Error bars represent SE; ** *p* < 0.01.

C57BL/6 mice with OSCC of the tongue, established through co-administration of 4-NQO and arecoline, were sacrificed, and the tumors were carefully separated into two parts. One part was processed for histopathology and it demonstrated characteristics of well-differentiated squamous cell carcinomas with keratinization and keratin-pearl formation (Figure 1A). The other part was minced for culture, as illustrated in Figure S1A. Two mouse OSCC cell lines, M1-2 and M2-3, were established from two separate mouse tumor specimens. The tumor cells attached tightly to the culture dish and showed typical squamous cell morphology. The M1-2 and M2-3 primary culture showed a heterogeneous cell population at the P2 stage. After continuous passages, most of the M1-2 cells at P21 stage grew with typical cobblestone-like epithelioid morphology (Figure 1B). Similarly, the M2-3 cells developed a typical cobblestone-like epithelioid morphology with tight adhesion at the P14 stage (Figure 1B). PCR detection with species-specific Cox I primers confirmed the murine origin of the M1-2 and M2-3 cells (Figure S1B).

The detection of short tandem repeat (STR) markers confirmed the B6 (C57BL/6Jnarl) mouse strain origin of both cell lines (Table S1). Distinctive spindle-shaped and polygonal cells were observed in the M1-2 and M2-3 cultures, respectively (Figure 1C). The cells retained similar morphological compositions for over 50 passages. Immunostaining with antibodies against epithelial antigens, such as pan-cytokeratin (pan-CK, Figure 1C) and the lack of expression of α-smooth muscle actin (α-SMA), ascertained the cells’ epithelial nature (Figure S2A) [17]. Epidermal growth factor receptor (EGFR) has been reported to be expressed in human cancers of epithelial origin [18]. M1-2 and M2-3 cells both demonstrated cytoplasmic and nuclear EGFR expression (Figure 1C). By MTS cell proliferation assay, the cell growth rate was higher in M2-3 cells than in M1-2 cells (Figure 1D).

### 2.2. Tumor Growth of Mouse OSCC Cells in Immunocompromised Mice and Syngeneic Mice

**Figure 2:**
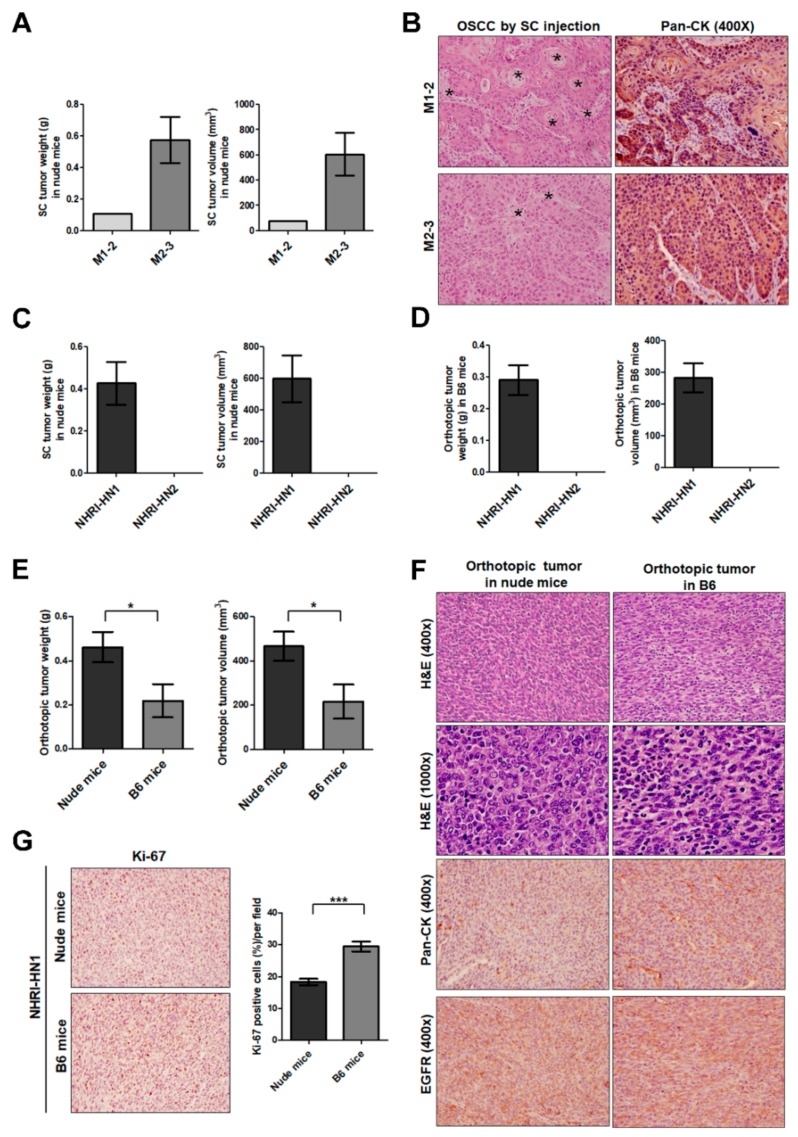
Tumor growth of M1-2 and M2-3 cells in nude mice and B6 mice. (**A**) Quantification of tumor weights (left panel) and volumes (right panel) in nude mice subcutaneously injected with 10^6^ M1-2 (*n* = 3) or M2-3 cells (*n* = 3) and sacrificed at 98 days post-inoculation. (**B**) Histological examination of the subcutaneous xenografts in nude mice with H&E staining (left panel) and IHC with anti-pan CK (right panel) at 400× magnification. Asterisks (*) indicate representative keratinization and keratin-pearl formation. (**C**) Quantification of tumor weights (left panel) and volumes (right panel) in nude mice subcutaneously injected with 10^6^ NHRI-HN1 (*n* = 4) or NHRI-HN2 cells (*n* = 4) and sacrificed at 42 days post-inoculation. (**D**) Measurement of the tumor weights (left panel) and volumes (right panel) in B6 mice orthotopically injected with 5 × 10^5^ NHRI-HN1 (*n* = 20 in total for four independent experiments) or NHRI-HN2 cells (*n* = 3) and sacrificed at 36–40 days post-inoculation. (**E**) Quantification of tumor weights volumes in nude mice and B6 mice orthotopically injected with 5 × 10^5^ NHRI-HN1 cells (*n* = 7 in total for two independent experiments) and sacrificed at 40 or 32 days post-inoculation. (**F**) Histological examination of NHRI-HN1 tumors in nude mice and B6 mice with H&E staining (upper panels) at 400× and 1000× magnifications and IHC with anti-pan CK (middle panel) and EGFR (lower panel) at 400× magnification. (**G**) Representative Ki-67-labeled NHRI-HN1 tumors are shown in the left panel. The percentage of positive Ki-67 signals was determined in NHRI-HN1-generated tumors in nude mice and B6 mice. Error bars represent SE. * *p* < 0.05; *** *p* < 0.001.

After subcutaneous injection into nude mice, M1-2 and M2-3 cells developed into tumors with an efficiency of 33% (*n* = 3; 95% confidence interval (CI) 6–79%) and 67% (*n* = 3; 95% CI 21–94%) at 98 days post-injection, respectively (Table 1). The subcutaneous tumor weights were 0.11 g (*n* = 1) and 0.575 ± 0.145 g (*n* = 2) for mice receiving M1-2 and M2-3 cells, respectively (Figure 2A and Figure S3A). The tumor volumes were also larger in the M2-3-injected mice (Figure 2A). Hematoxylin and eosin (H&E)-stained histological sections of the heterotransplanted subcutaneous tumors demonstrated the characteristics of well-differentiated squamous cell carcinoma with keratinization (Figure 2B). Additionally, the subcutaneous tumors of M1-2 and M2-3 cells showed strong pan-CK staining by immunohistochemistry (IHC), which confirmed epithelial carcinoma characteristics (Figure 2B). However, the M1-2 and M2-3 cells failed to develop any tumors after orthotopic injection into the oral cavity of syngeneic B6 mice (*n* = 3) by at least three months post-inoculation (Table 1).

### 2.3. Tumor Growth of In Vitro Selected Mouse OSCC Cells in Immunocompromised and Immunocompetent Mice

Two sublines enriched for more tumorigenic cells, NHRI-HN1 and NHRI-HN2, were established from M1-2 and M2-3 cells, respectively, through in vitro selection by sphere culture (Figure S2B). At 42 days post-subcutaneous injection into immunodeficient mice, only NHRI-HN1 cells developed into tumors, with an average weight of 0.4275 ± 0.1014 g (*n* = 4; Table 1, Figure 2C and Figure S3B). After the orthotopic injection of NHRI-HN1 and NHRI-HN2 cells into the oral cavity of B6 mice, only NHRI-HN1 cells generated tumors, with an average weight of 0.291 ± 0.04704 g (*n* = 20; Table 1, Figure 2D and Figure S3C).

In another set of experiments, NHRI-HN1 cells developed into orthotopic tumors with an average weight of 0.4614 ± 0.0688 g (*n* = 7) and 0.2184 ± 0.075 g (*n* = 7) in nude mice and B6 mice, respectively (Figure 2E and Figure S3D–E). H&E stained tissue sections of the orthotopic tumors of NHRI-HN1 cells demonstrated characteristics of sarcomatous differentiation, including the presence of pleomorphic spindle tumor cells and occasional multinucleated giant cells (Figure 2F). The expression of pan-CK and EGFR was also detected in the orthotopic tumors (Figure 2F). The tumors developed in B6 mice, although smaller, have more Ki-67-positive cells (29.46 ± 1.526%) when compared to those in nude mice (18.35 ± 0.9975%; Figure 2G). Our data suggested that immune response plays a suppressive role in tumor growth of NHRI-HN1 cells in syngeneic mice.

### 2.4. Effects of Immune Modulation on Syngeneic OSCC Tumors

**Figure 3:**
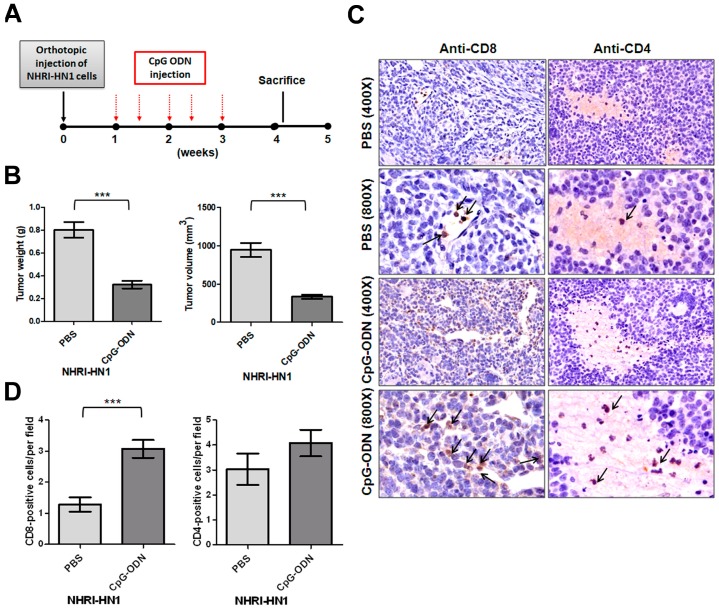
Effects of CpG-ODN on tumorigenesis of NHRI-HN1 cells. (**A**) The diagram shows the orthotopic allograft and CpG-ODN (100 μg/per injection) treatment scheme. (**B**) Quantification of tumor weights (left panel) and volumes (right panel) in the syngeneic mice orthotopically injected with NHRI-HN1 cells and treated either with phosphate buffered saline (PBS) or CpG-ODN (*n* = 6). (**C**) Immunohistochemistry (IHC) examination of NHRI-HN1 tumors in B6 mice treated with PBS or CpG-ODN using anti-CD8 and anti-CD4 antibodies (400× and 800× magnifications). (**D**) Quantification of the CD8- or CD4-positive cells/per field in NHRI-HN1 tumors treated with PBS or CpG-ODN. Error bars represent SE; *** *p* < 0.001.

We performed peritumoral injection of an oligonucleotide containing immunostimulatory CpG motifs (CpG-ODN). CpG-ODN signals through toll-like receptor 9 to stimulate both innate and adaptive immune responses to eliminate tumor cells to understand whether host immune response affects NHRI-HN1 cell-derived orthotopic tumor development [19]. The weight and volume of orthotopic tumors in mice receiving CpG-ODN injection were markedly reduced as compared to those injected with phosphate buffered saline (PBS) following the scheme that is shown in Figure 3A (*n* = 6, *p* < 0.001; Figure 3B and Figure S3F). Histological examination of tumors that were treated with CpG-ODN revealed a significant increase in the number of tumor-infiltrating CD8^+^ T cells (Figure 3C,D). No substantial difference in the numbers of infiltrating CD4^+^ cells was found between CpG-ODN-treated and control tumors (Figure 3C,D). These results suggest that the NHRI-HN1 cell-generated syngeneic model is suitable for investigating the effectiveness of immunotherapy for head and neck cancers.

### 2.5. Epithelial Mesenchymal Transition, Migration and Invasion in NHRI-HN1 Cells

**Figure 4:**
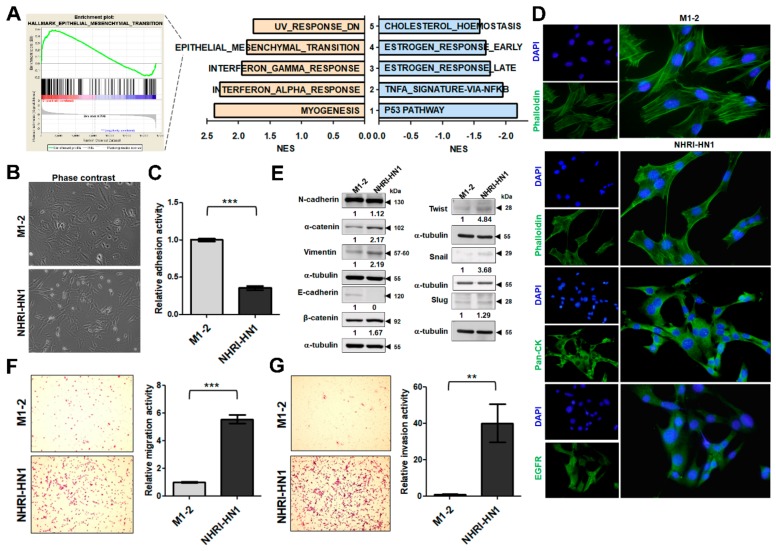
Detection of EMT and increased motility in NHRI-HN1 cells. (**A**) Summary of the most enriched pathways associated with tumorigenesis in syngeneic mice by comparing tumorigenic NHRI-HN1 cells with nontumorigenic cells, including M1-2, M2-3 and NHRI-HN2, using GSEA analysis. Blue indicates a negative normalized enrichment score (NES) and orange indicates a positive NES. (**B**) Morphology of M1-2 and NHRI-HN1 cells via phase-contrast microscopy at 100× magnification. (**C**) Relative adhesion activity in M1-2 and NHRI-HN1 cells, determined by normalizing the mean OD 490 nm value of NHRI-HN1 cells to that of M1-2 cells. (**D**) Cells stained with Alexa Fluor 488 phalloidin, anti-pan-CK and anti-EGFR antibodies at 400× magnification. (**E**) Immunoblot analysis of epithelial (E-cadherin, α-catenin and β-catenin), mesenchymal (N-cadherin and Vimentin) proteins and EMT-related transcription factors, including Twist, Snail and Slug in M1-2 and NHRI-HN1 cells. Protein levels were normalized to an internal control, α-tubulin. Ratios were determined by dividing the normalized protein levels in NHRI-HN1 cells by that in M1-2 cells. (**F**) Representative images (left) and relative data (right) for migration activity of M1-2 and NHRI-HN1 cells. (**G**) Representative images (left) and relative data (right) for invasion activity of M1-2 and NHRI-HN1 cells at 200× magnification. The relative migration or invasion activity was determined by normalizing the mean number of cells that have migrated or invaded per field of NHRI-HN1 cells to that of M1-2 cells. Error bars represent SE; ** *p* < 0.01; *** *p* < 0.001.

We performed gene expression analysis between tumorigenic NHRI-HN1 cells and non-tumorigenic cells, including M1-2, M2-3, and NHRI-HN2 cells, to identify the hallmark pathways related to the tumorigenic potential of NHRI-HN1 cells in syngeneic mice. The dataset is available in GEO/GSE139282. The principal component analysis (PCA)-based clustering assessment showed that NHRI-HN1 cells were slightly separated from the cluster of non-tumorigenic cells, similar to human OSCC and adjacent non-tumorigenic tissues (GSE37991; Figure S4A,B). We found myogenesis, interferon-alpha, interferon-gamma, epithelial mesenchymal transition (EMT), and down-regulated UV response highly expressed in NHRI-HN1 cells by gene set enrichment analysis (GSEA). The p53 pathway, tumor necrosis factor-alpha (TNF-α) signature via nuclear factor kappa-light-chain-enhancer of activated B cells (NF-κB), estrogen late response, estrogen early response, and cholesterol homeostasis were down-regulated when compared to non-tumorigenic cells (Figure 4A). Interestingly, the pathways that were enriched in differentially expressed genes when comparing NHRI-HN1 cells and non-tumorigenic cells resembled those that were differentially expressed when comparing human OSCC tissues and adjacent noncancerous tissues (Figure S4C). Both analyses demonstrated up-regulated interferon response and EMT pathways, as well as down-regulated lipid metabolism, supporting NHRI-HN1 cells as a reliable model for the study of human oral cancers.

We examined the morphology of M1-2 and NHRI-HN1 cells to explore the effects of marked upregulation of EMT signature genes in tumorigenic NHRI-HN1 cells and human OSCC tissues (Figure S4D,E). These cell types are morphologically similar under the phase-contrast microscope (Figure 4B), but the NHRI-HN1 cells displayed less adhesiveness than M1-2 cells by adhesion assay (Figure 4C). Phalloidin staining revealed a more mesenchymal-like actin filament organization in NHRI-HN1 cells (Figure 4D). Immunostaining with anti-pan-CK and EGFR antibodies, with notable EGFR staining (Figure 4D) and the absence of α-SMA expression (Figure S2A) confirmed the carcinoma-like nature of NHRI-HN1. Immunoblot analysis showed decreased E-cadherin (an epithelial cell marker) and increased Vimentin (a mesenchymal cell marker) expression in NHRI-HN1 cells when compared to M1-2 cells (Figure 4E). NHRI-HN1 cells demonstrated increased expression of EMT-related transcription factors, including Twist and Slug, as well as enhanced migration and invasion activities as compared to the parental M1-2 cells, as assessed by transwell migration assays (Figure 4E–G). Our data indicate that EMT had occurred in the NHRI-HN1 cells.

### 2.6. Cell Growth and Cancer Stemness Characteristics of NHRI-HN1 Cells

**Figure 5:**
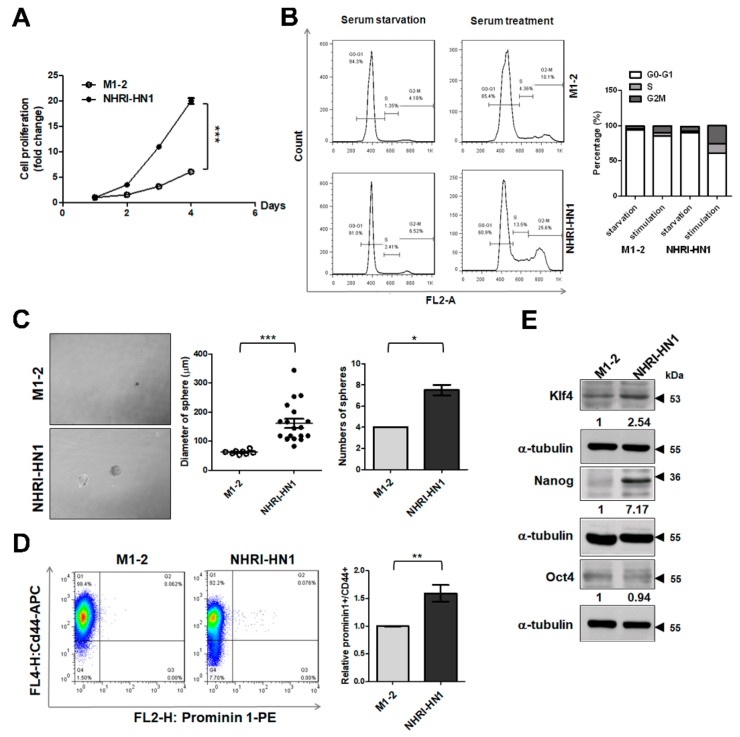
Analysis of cell growth and sphere formation in M1-2 and NHRI-HN1 cells. (**A**) Representative growth curves for M1-2 and NHRI-HN1 cells analyzed by MTS assay. The results were expressed as the fold change to the results at day 1. (**B**) Cell cycle analysis of M1-2 and NHRI-HN1 cells by Fluorescence Activated Cell Sorting (FACS). (**C**) Sphere-forming activity of M1-2 and NHRI-HN1 cells was assessed by sphere culture. Left: The presentative pictures for sphere formation at 50× magnification. Right: The average sphere diameter and number of spheres are shown. (**D**) Analysis of CSCs in M1-2 and NHRI-HN1 cells by FACS using anti-Prominin-1 and anti-CD44 antibodies. Representative data are shown in the left panel. The relative level of Prominin-1^+^/CD44^+^ cells is shown in the right panel. The Prominin-1^+^/CD44^+^ ratio was calculated by normalizing the mean percentage of Prominin-1^+^/CD44^+^ NHRI-HN1 cells to that of M1-2 cells. (**E**) Immunoblot analysis of stemness-related transcription factors Klf4, Nanog, and Oct4. The protein levels were normalized to internal control (α-tubulin). The ratios were calculated by dividing the normalized protein levels of NHRI-HN1 cells by those of M1-2 cells. Error bars represent SE; * *p* < 0.05; ** *p* < 0.01; *** *p* < 0.001.

NHRI-HN1 cells have a higher growth rate than M1-2 cells (Figure 5A). Consistently higher percentages of NHRI-HN1 cells in G2/M phase of the cell cycle were detected 24 h after serum stimulation (Figure 5B). The sphere formation assay showed that NHRI-HN1 cells formed spheroids more efficiently (greater size and number of spheres) than M1-2 cells (Figure 5C). Prominin-1 (CD133) and CD44 are stemness-related markers found in a variety of human tumors, including oral cancer, and the expression of these markers has been associated with the survival and prognosis of OSCC patients [20,21,22]. Fluorescence-activated cell sorter (FACS) analysis using anti-Prominin-1 and anti-CD44 showed that 0.075% of Prominin-1^+^/CD44^+^ cells were detected in NHRI-HN1 cells, whereas only 0.062% of double-positive cells were observed in the M1-2 cells (Figure 5D). We also analyzed the expression of stemness-related transcription factors, such as Klf4, Nanog, and Oct4 [23], and found an elevated expression of Klf4 and Nanog in NHRI-HN1 cells as compared to M1-2 cells (Figure 5E).

### 2.7. Inhibition of ERK Activation Impairs Cancer Stemness and Migration in NHRI-HN1 Cells

**Figure 6:**
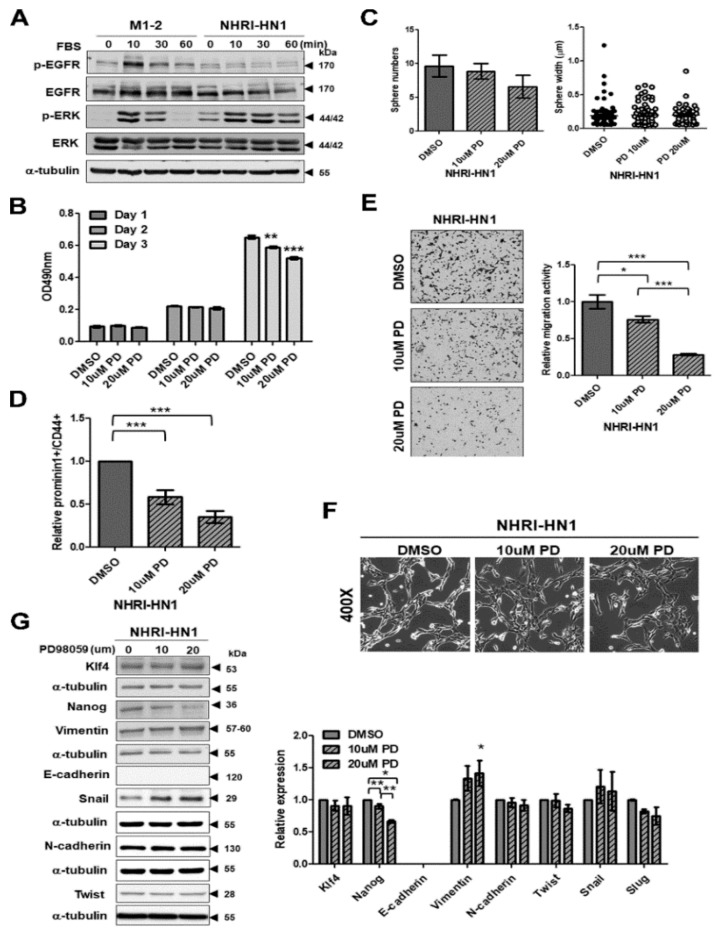
Inhibition of extracellular signal-regulated kinase (ERK) activation decreased cell migration and stemness in NHRI-HN1 cells. (**A**) Time course immunoblot assay for total and phosphorylated EGFR, ERK and AKT in M1-2 and NHRI-HN1 cells after 10, 30, and 60 min of fetal bovine serum (FBS) treatment following serum starvation. (**B**) Cell growth in PD98059-treated NHRI-HN1 cells analyzed by MTS assay for three days. (**C**) Sphere-forming activity in PD98059-treated NHRI-HN1 cells assessed by sphere culture. The average number and diameter of spheres are shown. (**D**) Analysis of CSCs in PD98059-treated NHRI-HN1 cells by FACS using anti-Prominin-1 and anti-CD44 antibodies. The relative Prominin-1^+^/CD44^+^ ratio was calculated by normalizing the mean percentage of Prominin-1^+^/CD44^+^ in PD98059-treated NHRI-HN1 cells to that of untreated cells. (**E**) Representative data show the relative migration activity of NHRI-HN1 cells treated with PD98059. Left panel: Cells that have migrated (100× magnification). Right panel: Relative migration activity was calculated by normalizing the mean number of PD98059-treated cells per field that have migrated to that of control cells. (**F**) Morphology of NHRI-HN1 cells treated with PD98059 (400× magnification). (**G**) Left panel: Immunoblot analysis of Klf4, Nanog, E-cadherin, N-cadherin, Vimentin, Twist and Snail proteins from NHRI-HN1 cells treated with PD98059. Right panel: Total protein levels were normalized to internal control (α-tubulin), and ratios were calculated relative to that the specific protein levels of control cells. Error bars represent SE; * *p* < 0.05; ** *p* < 0.01; *** *p* < 0.001.

Next, we investigated possible intracellular signaling pathways contributing to the differences between M1-2 and NHRI-HN1 cells. We found minimal differences in levels of the phospho- serine/threonine kinase AKT, phospho-NF-kB p65, phospho-signal transducer and activator of transcription 3 (p-Stat3), and phospho-Src between M1-2 and NHRI-HN1 cells (Figure S5A). The greatest differences between M1-2 and NHRI-HN1 cells were detected in levels of phosphorylated EGFR (p-EGFR) and phosphorylated ERK (p-ERK Thr202/Tyr204) (Figure 6A). EGFR activation by serum stimulation occurred in M1-2 cells, while EGFR was constitutively dephosphorylated in the NHRI-HN1 cells (Figure 6A). We sequenced EGFR cDNA synthesized from NHRI-HN1 cells to determine whether EGFR mutation was associated with constitutive dephosphorylation. No mutation was found. The EGFR inhibitor gefitinib consistently blocked the recombinant epidermal growth factor (EGF)-induced phosphorylation of EGFR and AKT, indicating that the EGF-EGFR-AKT signaling was intact in NHRI-HN1 cells, although with reduced sensitivity (Figure S5B). Gefitinib partially inhibited EGF-induced ERK phosphorylation, but it had no effect on the basal level of ERK phosphorylation in NHRI-HN1 cells (Figure S5B).

Additionally, the basal level of ERK phosphorylation was higher in NHRI-HN1 cells than in M1-2 cells under serum starvation (Figure 6A), which indicated constitutively active ERK in NHRI-HN1 cells. ERK activation was also demonstrated in some human OSCC cells, including OC3, OEC-M1, TW2.6, and SAS, as compared to dysplastic oral keratinocytes (DOKs) (Figure S6A). The mitogen-activated protein kinase (MAPK) kinase inhibitor PD98059 suppressed ERK phosphorylation in NHRI-HN1 cells (Figure S5C) and it had slight inhibitory effects on cell growth of NHRI-HN1 cells on day 3 (Figure 6B), while PD98059 treatment inhibited the number of NHRI-HN1 cell spheres (Figure 6C). The relative amounts of Prominin-1^+^/CD44^+^ cells were significantly suppressed in PD98059-treated NHRI-HN1 cells (Figure 6D), and the migration activity of NHRI-HN1 cells was markedly diminished by PD98059 treatment in a dose-dependent manner (Figure 6E). However, PD98059 treatment did not lead to changes in the shape of NHRI-HN1 cells when compared to untreated controls (Figure 6F). Immunoblot analysis showed decreased Nanog expression in PD98059-treated NHRI-HN1 cells as compared to the untreated cells, but no differences in the expression of the other stemness- and EMT-related proteins assessed (Figure 6G). The blocking ERK activation in human SAS and OEC-M1 cells did not affect cell growth (Figure S6B), but it did suppress sphere formation (Figure S6C) and cell motility (Figure S6D). Our data indicate that ERK activation is required for enhanced cancer stemness and the migration of NHRI-HN1 and human OSCC cells.

## 3. Discussion

Syngeneic mouse tumor models are historically and currently the most commonly utilized preclinical models for the evaluation of anticancer therapies. As these models are fully immunocompetent, they are particularly useful for testing immuno-oncology agents. They can also be easily used to study de novo antitumor immune responses without adoptive transfer of immune cells [24]. There has been limited availability of syngeneic mouse OSCC cell lines [10,12], and those available lack the context of tumors developed by East and Southeast Asian patients with habits of betel quid chewing and smoking. We report the establishment and characterization of a novel murine cell line, NHRI-HN1, derived from spontaneously developed arecoline/4-NQO-induced oral cancers, which can be used to generate a syngeneic mouse model for OSCC.

We established two mouse OSCC cell lines with different tumorigenic capacity from mice with arecoline/4-NQO-induced oral and esophageal cancers [25,26]. Nude mice that were injected with either M1-2 or M2-3 cells developed tumors, but tumors were not developed after cell injection in immunocompetent B6 mice (Table 1), which suggests tumor cell elimination by the host immunity. After in vitro selection of M1-2 and M2-3 cells using sphere culture, we derived one cell line, NHRI-HN1, which is competent in forming tumors in B6 mice (Table 1 and Figure 2D,E). Two known mechanisms for cancer immune evasion are the selection of mutant cells resistant to immune effectors, and the progressive formation of an immunosuppressive environment within the tumor [27]. No tumor developed in mice with orthotopic injection of alternate cell line NHRI-HN2 (Figure 2D), which suggested that NHRI-HN2 clones were destroyed by host immune cells or lost their tumorigenicity after in vitro selection. In contrast, NHRI-HN1 cell allograft produces an ideal syngeneic model for investigating factors contributing to OSCC tumor persistence in the context of a normal immune system.

Baines et al. reported CpG-ODN-induced immune-mediated tumor regression in a murine model of cervical carcinoma. The tumor regression and extended survival that were induced by CpG-ODN treatment required the participation of CD8^+^ T cells [28]. We were also able to use CpG-ODN injection to suppress tumor growth in our syngeneic model (Figure 3B and Figure S3F), demonstrating that tumor regression correlates with increased CD8^+^ T cell infiltration of tumors (Figure 3C,D), and successful conversion of a “cold” tumor into a “hot” one by CpG-ODN. These results highlight the advantage of NHRI-HN1-generated syngeneic models in the evaluation of immuno-oncology agents. NHRI-HN1 cells can also be further genetically manipulated to evaluate the role of biomarkers of tumor-specific intrinsic sensitivity or resistance in response to immunotherapy [24].

NHRI-HN1 cells demonstrated an up-regulation of interferon signaling and EMT pathway components, and down-regulation of lipid metabolism when compared to nontumorigenic cells, similar to the tumorigenesis of human OSCC (Figure 4A and Figure S4C). Yang et al. reported that primary OSCC expressing a hallmark EMT signature, low E-Cadherin, and high Vimentin has a significant increase in the satellite’s average distance when compared to those without an EMT signature [29], indicating that NHRI-HN1 cells with a strong EMT signature might have greater potential to drive cancer progression. EMT has long been recognized as a major player in cancer progression and metastasis, and its characteristic protein expression has been well-investigated in oral tumorigenesis [30]. We demonstrated that aggressive NHRI-HN1 cells showed EMT characteristics to confirm the up-regulation of the EMT signature (Figure 4D–G).

The dramatic increase in tumorigenicity of NHRI-HN1 cells in syngeneic mice, as compared to the parental M1-2 cells (Table 1), might have resulted from selective enrichment of a highly malignant subpopulation of M1-2 cells. NHRI-HN1 cells also demonstrated more sphere formation activity and a higher percentage of Prominin-1^+^/CD44^+^ cells as compared to the parental M1-2 cells (Figure 5C,D). Moreover, the expression levels of stemness-related proteins, including Nanog and Klf4 [31], were increased in NHRI-HN1 cells, implicating these transcription factors in the regulation of gene networks that are associated with self-renewal and the differentiation of CSCs (Figure 5E).

EGFR overexpression was identified in OSCC patients in Taiwan [32], and was also confirmed in a mouse model that was developed via arecoline and 4-NQO co-administration [25]. EGFR expression was readily detectable in the tumors that formed by NHRI-HN1 cells in both nude mice and B6 mice (Figure 2F). Therefore, the syngeneic model that was developed while using NHRI-HN1 cells represents a clinically relevant system that recapitulates the environmental factor-induced molecular events leading to tumorigenesis in East and Southeast Asian OSCC patients. No significant difference in EGFR expression was found between the NHRI-HN1 and M1-2 cells in culture (Figure 6A). EGFR gene sequencing and gefitinib inhibition of EGF-induced signaling (Figure S5B) also suggested that the EGFR signaling pathway is intact in NHRI-HN1 cells. The reduction in serum-stimulated EGFR phosphorylation in NHRI-HN1 cells might be due to negative feedback-based regulation of constitutive ERK activation, therefore reducing EGFR phosphorylation [33] (Figure 6A).

The overexpression and activation of ERK are frequently detected in many types of cancers, including OSCC [34]. ERK activation was found in NHRI-HN1 cells and human OSCC cells, similar to a previous study that reported ERK activation in aggressively growing mouse oral cancer cell lines (Figure 6A and Figure S6A) [12]. ERK activation drives cell proliferation, migration, and invasion through anti-apoptotic and proliferative signaling pathways [35,36], and ERK activation modulates cancer aggressiveness by the regulation of CD44 expression [12]. Similarly, we observed that blocking ERK activation suppressed sphere formation, CSC numbers, and migration activity in NHRI-HN1 cells (Figure 6C–E) and human OSCC cells (Figure S6B–D). ERK can also enhance metastasis by inducing EMT and expression of Slug and Snail [37], yet ERK inhibition in NHRI-HN1 cells induced neither consistent effects on expression of EMT markers nor obvious changes in cell morphology (Figure 6F,G). Our data indicate that ERK activation is only partially responsible for the tumorigenicity of NHRI-HN1 cells.

Additionally, we observed reduced Nanog expression in NHRI-HN1 cells after PD98059 treatment (Figure 6G), which suggested that Nanog plays a role in cancer stemness and the migration of NHRI-HN1 cells. Noh et al. reported that human cancer cells with high Nanog expression exhibit stem-like, anti-apoptotic properties, and they are resistant to immune attack [38]. The inhibition of Nanog in a murine model of colon cancer rendered tumor cells sensitive to immune-mediated clearance and led to successful long-term control of the disease [39]. In concordance with the literature, our data support a possible role for Nanog in OSCC cell EMT, cancer stemness, and immune evasion.

Overall, our newly-developed mouse cell line demonstrates histological features and pathway signatures similar to human OSCC tissues and it provides a potential platform for the investigation of novel combinations of chemotherapy, targeted therapy, and immunotherapy. A pivotal role for ERK activation in modulating cell migration and stemness was discovered in this novel murine OSCC cell line, similar to human OSCC cell lines. Our findings from the characterization of a novel syngeneic model of OSCC indicate links between immune selection, mobility, and disease progression in the stem-like tumor phenotype.

## 4. Materials and Methods

### 4.1. Ethics Statement

All of the animal studies followed the procedures for the Care and Use of Laboratory Animals of National Health Research Institutes, Taiwan. The Institutional Animal Care and Use Committee of National Health Research Institutes approved the protocols (Protocol No.: NHRI-IACUC-107011-A and 108017-A).

### 4.2. Mouse Tongue Squamous Cell Carcinoma

The mouse tongue squamous cell carcinoma was induced with 4-nitroquinoline 1-oxide (4-NQO) and arecoline, as described previously [25,26]. Briefly, the mice (C57BL/6JNar1) were treated with 200 μg/mL of 4-NQO (Sigma-Aldrich, St. Louis, MO, USA) and 500 μg/mL of arecoline hydrobromide (Fluka, Buchs, Switzerland) in drinking water for 16 weeks and fed with normal drinking before being sacrificed in the end of seven months and the tumors on their tongues were carefully separated into two parts. One part was processed for histopathological examination after 10% buffered formalin fixation and stained with hematoxylin and eosin (H&E, Sigma-Aldrich). The other part was excised on a clean bench and cut into pieces. The cells were collected with phosphate buffered saline (PBS, Sigma-Aldrich), centrifuged and cultured in Dulbecco’s modified Eagle medium (DMEM, Invitrogen/Thermo Fisher Scientific, Waltham, MA, USA) with 10% fetal bovine serum (FBS, Gibco/Thermo Fisher Scientific) at 37 °C in an incubator that was supplied with 5% CO_2_. When the cell confluence reached 80%, detaching cells while using 0.05% trypsin passaged the cells. After repeated passages, stably growing tumor cells were obtained and stored.

### 4.3. In Vitro Selection and Cell Culture

The cells (1 × 10^4^) were incubated with Dulbecco’s modified Eagle medium (DMEM, Invitrogen/Thermo Fisher Scientific)/F12 medium (Sigma-Aldrich) containing with 100× N2 supplement (Gibco/Thermo Fisher Scientific), 20 ng/mL epidermal growth factor (EGF, Abcam, Cambridge, UK), and B27 supplement (Gibco/Thermo Fisher Scientific) for two weeks. The tumor spheres were collected and stably cultured in dishes with DMEM with 10% fetal bovine serum (FBS, Gibco/Thermo Fisher Scientific) at 37 °C in an incubator supplied with 5% CO_2_. All of the mouse oral cancer cell lines were established before November 2016, authenticated by amplification of Cox I and short tandem repeat analysis in June, 2017, and then kept free of mycoplasma contamination. Human OSCC cells, including DOK, as established from a human dysplastic oral mucosa [40], CGHNC9 established from an oral cancer patient [41], OC3 established from an OSCC specimen [42], OEC-M1 established from an oral epidermoid carcinoma [43], TW2.6 established from a buccal carcinoma, and SAS established from a poorly differentiated tongue squamous cell carcinoma [44], were maintained within 20 passages, as described previously [45]. Human OSCC cells were authenticated while using short tandem repeat analysis that was performed by Center for Genomic Medicine, National Cheng Kung University, Tainan, Taiwan and Mission Biotech, Taipei, Taiwan before June 2018.

### 4.4. DNA Extraction and Amplification of Cox I and Short Tandem Repeat Sequences With Polymerase Chain Reaction

Confirmation of mouse cell lines by a polymerase chain reaction (PCR)-based method [46,47]. Genomic DNA was prepared while using a genomic DNA extraction kit, according to the manufacturer’s instructions (Qiagen, Valencia, CA, USA). The yield and purity of DNA were analyzed using a NanoDrop 1000 spectrophotometer (NanoDrop Technologies, Wilmington, DE, USA). Confirmation of mouse origin by PCR used the following primers: human COX I-F: 5′ TTCGGCGCATGAGCTGGAGTCC; human COX I-R: 5′ TATGCGGGGAAACGCCATATCG; mouse Cox I-F: 5′ ATTACAGCCGTACTGCTCCTAT; D9S171-REV: 5′ GTGTCTTACCCTAGCACTGATGGTATAGTCT, D9S171-NED: 5′AGCTAAGTGAACCTCATCTCTGTCT; mouse Cox I-R: 5′ CCCAAAGAATCAGAACAGATGC; mouse 15-3-F: 5′ TCTGGGCGTGTCTGTCATAA, mouse 15-3-R: 5′ GTTCTCAGGGAGGAGTGTGCT. For amplification, 20 μL of reaction mixture contained 1μM of each primer, 200 μM dNTP, and 2.5U KOD FX DNA polymerase (Toyobo CO., LTD., Osaka, Japan) in PCR buffer and heated to 95 °C for 5 min, followed by 30 cycles of denaturation at 95 °C for 30 s, primer annealing at 55 °C for 30 s, and extension at 72 °C for 1 min. In the last cycle, the extension step was prolonged to 5 min. The amplification product was run on 2% agarose gel, stained with ethidium bromide (Invitrogen), visualized under UV light, and then photographed. The sequences for ten short tandem repeat primers used in this study were published with modifications, as shown previously [47]. Amplification was conducted in a 10 μL final volume while using the reaction conditions recommended by the manufacturer’s instructions (Toyobo). Capillary electrophoresis was conducted on a 3700 Genetic Analyzer (Applied Biosystems, Foster City, CA, USA). Allele designation was derived by comparing sample peaks by allelic ladder peaks using the Gene Mapper 3.5 software (Applied Biosystems).

### 4.5. Immunohistochemistry

IHC was conducted as described previously [48]. Following primary antibodies were used: anti-pan cytokeratin (1:200, pan-CK, NB120-6401, Novus biological, Littleton, CL, USA), anti-epidermal growth factor receptor (1:30, EGFR, sc-03, Santa Cruz, Santa Cruz, CA, USA), and anti-Ki-67 (1:500, NCL-Ki-67p, Novocastra laboratories, Newcastle upon Tyne, UK).

### 4.6. Morphological Analysis

Exponentially growing cells were examined under inverted phase-contrast microscope, as previously described [48]. The cultured cells on coverslips were fixed, as previously described [49]. After staining with Alexa Fluor 488 phalloidin (1:1000, Molecular Probes, Ungene, OR, USA), the coverslips were mounted on slides and then examined while using a fluorescent microscope.

### 4.7. Immunofluorescence

10^5^ cells were seeded onto cover slides in six-well plate. The next day, the cells were rinsed with Dulbecco’s Phosphate-Buffered Saline (DPBS, Thermo Fisher Scientific) and then fixed with 4% paraformaldehyde (Sigma-Aldrich) for 10 min. at room temperature, followed by permeabilization with 0.1% Triton X-100 (Sigma-Aldrich). The cells were stained with anti-pan CK (1:100, NB120-6401, Novus biological), anti-EGFR (1:30, sc-03, Santa Cruz) or anti-α-smooth muscle actin (α-SMA, 1:200, ab5694, Abcam) antibody overnight at 4 °C. The cells were then washed with DPBS three times for 10 min. per wash and then incubated with DyLight 488-labeled (1:500, 611-141-002, Thermo Fisher Scientific) or DyLight 549-labeled secondary antibody (1:500, 611-142-002, Thermo Fisher Scientific) at room temperature for one hour. After washed with DPBS three times for 10 min each, cover slips were counterstained with 4′,6-diamidino-2-phenylindole (DAPI, Vector Lab, Burlingame, CA, USA), mounted and viewed under fluorescent microscopy.

### 4.8. Cell Proliferation

The growth curves were obtained as described previously [50]. Briefly, 10^3^ cells/well were plated in 96-well plates and Cell-Titer 96 Aqueous Non-radioactive Cell Proliferation assay measured cell growth (Promega, Madison, WI, USA) for 4–5 days based on the manufacturer’s instructions. The cell growth curves were determined by calculating the mean value of absorbance at 490 nm while using a 96-well plate reader. The results were expressed as the fold change relative to the result at day 1.

### 4.9. Subcutaneous Tumor Growth In Nude Mice

Subcutaneous tumor growth assay was conducted, as described previously [51]. Five to six-week-old male nude mice (BALB/cAnN.Cg-Foxn1^nu^/CrlNarl) were purchased from National Laboratory Animal Center (NLAC, Taiwan). 10^6^ cells that were suspended in sterile PBS were subcutaneously injected into either left or right flank of the same mice. The tumors were measured weekly and tumor volume (mm^3^) was calculated using the formula 1/2 × (length) × (width)^2^ to obtain the tumor growth curve. The mice were sacrificed at 98 days post-injection of M1-2 or M2-3 cells (*n* = 3/group) and 42 days after injection of NHRI-HN1 or NHRI-HN2 cells (*n* = 4/group). The subcutaneous tumors were weighed, photographed, processed by the Pathology Core Lab (National Health Research Institutes, Miaoli, Taiwan), and their histopathology examined by H&E staining. In accordance with the definition of 3R (replacement, reduction, and refinement), as well as the guidelines for design and statistical analysis of experiments using laboratory animals, the in vivo analysis has been presented by averaging all obtained data [52]. The mice were randomized to the group assignment.

### 4.10. Orthotopic Injection in Syngeneic Mice

The procedures for orthotopic injection were described previously [36]. Briefly, 5–6-week-old male mice (C57BL/6JNar1) purchased from the National Laboratory Animal Center were anesthetized via the inhalation of 5% isoflurane (Piramal Critical Care, Bethlehem, PA, USA). 5 × 10^5^ M1-2 or M2-3 cells in 50 μL in sterile PBS were injected into the buccal mucosa of mice (*n* = 3/group) and mice were carefully monitored until sacrifice at least three months post-inoculation. 5 × 10^5^ of NHRI-HN1 or NHRI-HN2 cells in 50 μL in sterile PBS were injected into the buccal mucosa of mice (*n* = 4/ group). The mice were carefully monitored until they were sacrificed at 40 days post-inoculation. 5 × 10^5^ cells in 50 μL PBS were orthotopically inoculated into each mouse of three batches (*n* = 5–6) and mice were sacrificed at 36–38 days post-inoculation for the confirmation of tumor growth of NHRI-HN1 cells. The tumor growth curve was developed by measuring tumor dimensions and calculating volume (mm^3^) using the formula 1/2 × (length) × (width)^2^. The orthotopic tumors were weighed and then processed by the Pathology Core Lab.

### 4.11. Orthotopic Injection of NHRI-HN1 Cells in Nude Mice and Syngeneic Mice

5 × 10^5^ cells in 50 μL in sterile PBS were injected into the buccal mucosa of two batches of 5–6-week-old male nude mice (BALB/cAnN.Cg-Foxn1^nu^/CrlNarl) (*n* = 4 and *n* = 3 per batch, respectively) and C57BL/6JNar1 mice (*n* = 4 and *n* = 3 per batch, respectively) and mice were sacrificed at 40 and 32 days post-inoculation for first and second batches, respectively. The tumor growth curve was developed by measuring the tumor dimensions and calculating volume (mm^3^) while using the formula 1/2 × (length) × (width)^2^. The orthotopic tumors were weighed and then processed by the Pathology Core Lab.

### 4.12. CpG Oligodeoxynucleotide (CpG-ODN) Treatment

ODN obtained from Dr. Tsung-Hsien Chuang (Immunology Research Center, National Health Research Institutes, Taiwan). Phosphorothiolate-modified CpG-2722 (GTTGTCGTTTTTTGTCGTT) used in this study was synthesized by integrated DNA Technology (Singapore), and described in previous study [53]. After successful anesthesia, 2.5 × 10^5^ cells in 50 μL in sterile PBS were injected into the buccal mucosa of mice, which were then carefully monitored (*n* = 6/group). For CpG-ODN treatment, 100 μg of CpG-ODN dissolved in 50 μL of sterile PBS were injected into the same side of the oral cavity on days 7, 10, 14, 17, and 21 post-injection of NHRI-HN1cells, and the mice were sacrificed at 29 days post-inoculation. The tumor growth curve was obtained by measuring the tumor volume (mm^3^) with the formula 1/2 × (length) × (width)^2^. The Pathology Core Lab weighed and then processed the orthotopic tumors.

### 4.13. Gene Expression and Pathway Analysis

Total RNA was extracted while using Trizol reagent (Invitrogen/Thermo Fisher Scientific) and purified using RNeasy mini kit (QIAGEN). The RNA quality examined for OD260/280 greater than 1.8 using Nanodrop and RNA integrity number values greater than 7.0 using a Bioanalyzer 2100 (Agilent Technologies, Santa Clara, CA, USA). Genome-wide gene expression analysis was performed while using the Affymetrix Mouse Clariom S Assay (Affymetrix, Santa Clara, CA, USA), including 22,100 well-annotated genes, by the Microarray Core Laboratory of the National Health Research Institutes. Microarray analysis was conducted and followed the standard Affymetrix procedures. The hybridized slides were scanned by Affymetrix Gene Chip scanner 3000 7G system (Affymetrix, Santa Clara, CA, USA). Raw data were generated while using Partek Genomics Suite version 7 software (St Louis, MI, USA). Principal component analysis (PCA) and heatmap of EMT signature genes were generated using Python PCA package and R gplots package, respectively. Pathway analysis was performed using Gene set enrichment analysis and Hallmark pathway collection, as previously described [54,55].

### 4.14. Adhesion assay

Growing cells (1 × 10^6^) were seeded into 10 cm culture dishes and incubated overnight. Serum starvation began 12 h before the assay. The cells were detached with 10 mM EDTA for 10–15 min, washed with DMEM, and then resuspend in DMEM with 0.1% BSA. 2 × 10^5^ cells were plated in collagen I-coated 96-well plates and incubated at 37 degrees for 40 min, and then washed four times with DMEM. The cells in DMEM with 10% FBS were then incubated at 37 degrees for 4 h for recovery, followed by the MTS assay to determine cell density.

### 4.15. Immunoblot Assay

Immunoblot assay was performed as previously described [48]. Primary antibodies used: anti-Klf4 (1:1000, sc-20691, Santa Cruz), anti-Nanog (1:1000, GTX627421, GeneTex, Irvine, CA, USA), anti-Sox2 (1:3000, GTX101507, GeneTex,), anti-Oct4 (1:1000, GTX627419, GeneTex), anti-N-cadherin (1:1000, 610920, BD, Franklin Lakes, NJ, USA), anti-Vimentin (1:5000, MS-129-P0, Thermo Scientific), anti-E-cadherin (1:1000, 610182, BD), anti-β-catenin (1:1000, 610154, BD), anti-Twist (1:1000, sc-15393, Santa Cruz), anti-Snail (1:1000, #3895, Cell Signaling, Danvers, MA, USA), anti-Slug (1:1000, AP2053, ABGENT), anti-Src (1:1000, #2109, Cell Signaling), anti-phosphorylated Src (1:1000, #2101, Cell Signaling), anti-nuclear factor kappa-light-chain-enhancer of activated B cells (NF-κB, 1:1000, sc-8008, Santa Cruz), anti-phosphorylated NF-κB (1:1000, #3033, Cell Signaling), anti-protein kinase B/AKT (1:1000, #9272, Cell Signaling), anti-phosphorylated AKT (1:1000, #9271, Cell Signaling), anti-extracellular signal-regulated kinase (ERK, 1:5000, sc-94, Santa Cruz). anti-phospho-ERK (1:1000, sc-7383, Santa Cruz), anti-epidermis growth factor receptor (EGFR) (1:1000, sc-03, Santa Cruz), anti-phospho-EGFR (1:1000, #2234, Cell Signaling), anti-signal transducer and activator of transcription 3 (STAT3, 1:1000, GTX104616, GeneTex), anti-phospho-STAT3 (1:1000, #9131, Cell Signaling), and anti-α-tubulin (1:5000, MS-581-P0, Thermo Scientific). Second antibodies used: goat anti-mouse IgG (1:5000, 115-035-174, Jackson ImmunoResearch, West Grove, PA, USA) and goat anti-rabbit IgG (1:5000,111-035-003, Jackson ImmunoResearch). The uncropped scans of western blots were shown in Figure S7.

### 4.16. Migration and Invasion Assays

The assays were performed using transwells, as described previously [49]. Relative migration or invasion activity were determined by normalizing the mean number of cells that had migrated or invaded per field in the experimental condition to that of control cells.

### 4.17. Cell Cycle Analysis

Cells were serum-starved for 24 h and then cultured in growth medium containing 10% FBS for 24 h. The cells were trypsinized and fixed with 70% ethanol followed by staining with propidium iodide (Sigma-Aldrich). DNA content was determined by flow cytometry (FACSCalibur, BD) and FlowJo 7.6 software (FlowJo, Ashland, OR, USA).

### 4.18. Sphere Formation Assay

Spheroids were formed as described previously [56] and then examined under a bright-field microscope. The size and diameter of tumor sphere were quantitatively measured by ImageJ (National Institutes of Health, Bethesda, MD, USA).

### 4.19. Detection of Cancer Stem Cells by Fluorescence Activated Cell Sorting (FACS) Analysis

Cells were collected with 0.05% trypsin-EDTA solution and resuspended at 1 × 10^6^ cells per ml in 0.5% bovine serum albumin (BSA, Sigma-Aldrich) solution containing diluted anti-mouse promonin-1-PE (1:100, 130-102-834, Miltenyi Biotec, North Rhine-Westphalia, Germany) and anti-mouse CD44-APC antibody (1:100, 130-110-118, Miltenyi Biotec). For the identification of Prominin-1^+^/CD44^+^ population, the samples were analyzed while using a FACSCalibur flow cytometer (BD Biosciences) and FlowJo 7.6 software. The purity of sorted cells was more than 95% by additional flow cytometric analysis. A total of 10^5^ viable cells were collected.

### 4.20. EGFR Sequencing

The cDNA of EGFR was amplified by PCR using primers listed below with KOD FX DNA polymerase (Toyobo CO., LTD.). The first cycle of amplifications was performed using a 2 min. initial denaturation at 94 °C; followed by 30 cycles of 10 s at 98 °C, 30 s at 55 °C, and 2 min. at 68 °C; and, a 7 min. final extension at 68 °C. The final products were cleared and sequenced with the internal primers listed below using ABI PRISM 3730 DNA Analyzer (Applied Biosystems). Amplification primers: mEGFR-F1: 5′ ATGCGACCCTCAGGGACC; mEGFR-R1: 5′ AGTATTATTTCTAGGTTCTC; mEGFR-F2: CCCGAGAACTAGAAATTCT; mEGFR-R2: 5′ACCAGTACATTCCTGGCTGC; mEGFR-F3: 5′ TCATGCCCTACGGTTGCCT; mEGFR-R3: 5′ TCATGCTCCAATAAACTCAC. Sequencing primers: mEGFR-seq1: TGGGCACAGATGATTTTGGT; mEGFR-seq2: GTGCGATTC AGCAACAACC; mEGFR-seq4: CACACTGTGTCAAGACCT; mEGFR-seq6: CTACAGACTCCAACTTTTAC.

### 4.21. Statistical Analysis

The data were expressed as mean ± standard error of the mean (SE) and analyzed, as described previously [45]. Briefly, analysis was performed with GraphPad Prism version 5.01 (GraphPad Software, San Diego, CA, USA). Two-tailed student’s t-test was used to assess differences between two groups. The Wilson score interval method was used to determine the 95% confidence interval (CI) [57]. *p* < 0.05 was considered to be statistically significant for all comparisons.

## 5. Conclusions

Our newly developed mouse cell line demonstrates histological features and pathway signatures similar to human OSCC tissues from patients from East and Southeast Asia. Similar to the human OSCC cell lines, a pivotal role for ERK activation in modulating cell migration and stemness was discovered in this novel murine OSCC cell lines. NHRI-HN1 cells show tumorigenic characteristics of EMT, cancer stemness, and ERK activation appropriate for modeling human OSCC and provide a potential platform for investigation of treatments for use in combination with immunotherapy.

## Figures and Tables

**Table 1:** Tumor formation in immunocompromised and immunocompetent mice injected with mouse oral squamous cell carcinoma (OSCC) cells.

Cell Lines	Nude Mice (Subcutaneous Injection)		B6 Mice (Orthotopic Injection)	
	Tumors/No. of Mice	Tumorigenesis (%)	Tumors/No. of Mice	Tumorigenesis (%)
**M1-2**	1/3	33.33	0/3	0
**M2-3**	2/3	66.67	0/3	0
**NHRI-HN1**	4/4	100	3/3	100
**NHRI-HN2**	0/4	0	0/3	0

## Supplementary Materials

The following are available online at https://www.mdpi.com/2072-6694/12/1/61/s1. Table S1: Short tandem repeat (STR) genotyping of mouse cell lines, Figure S1: Establishment and genetic identification of mouse OSCC cells, Figure S2: Characterization of mouse OSCC cells, Figure S3: Tumor growth of mouse OSCC cells in nude mice and B6 mice, Figure S4: Gene expression analysis of 4 mouse OSCC cell lines and 40 pairs of OSCC and their corresponding adjacent nontumor tissues, Figure S5: ERK activation in NHRI-HN1 cells, Figure S6: Blockage of ERK activation by PD98059 inhibited cell migration and stemness of human OSCC cells. Figure S7: The uncropped scans of western blots from the main and supplementary figures.
