# Supplementary material for: ERK Activation Modulates Cancer Stemness and Motility of a Novel Mouse Oral Squamous Cell Carcinoma Cell Line
Source: Cancers (Basel). 2019 Dec 24;12(1):61. doi: 10.3390/cancers12010061 (PMC7016611; doi:10.3390/cancers12010061)
Supplement: Supplementary file 1 [file cancers-12-00061-s001.pdf]

cancers

Article

Supplementary Materials: ERK Activation Modulates Cancer Stemness and Motility of a Novel Mouse Oral Squamous Cell Carcinoma Cell Line

Table S1. Short tandem repeat (STR) genotyping of mouse cell lines.

STR markers	Expected base pair	B6	M1-2	NHRI-HN1	M2-3	NHRI-HN2
18-3	150	152.06	152.26	151.98	152.08	151.98
4-2	237	236.5	236.53	236.55	236.52	236.49
6-7	347	353.96	353.95	353.97	354.08	353.98
9-2	228	232.06	232.01	231.92	231.78	231.82
15-3	196	200.37	200.37	200.38	200.38	200.28
6-4	294	298.09	298.01	298.02	298.09	297.81
12-1	224	227.89	227.85	227.96	227.73	227.59
			231.89	232.03		
5-5	341	343.44	343.45	343.51	343.28	343.25
x-1	402	406.18	406.17	406.28	406.18	406.32

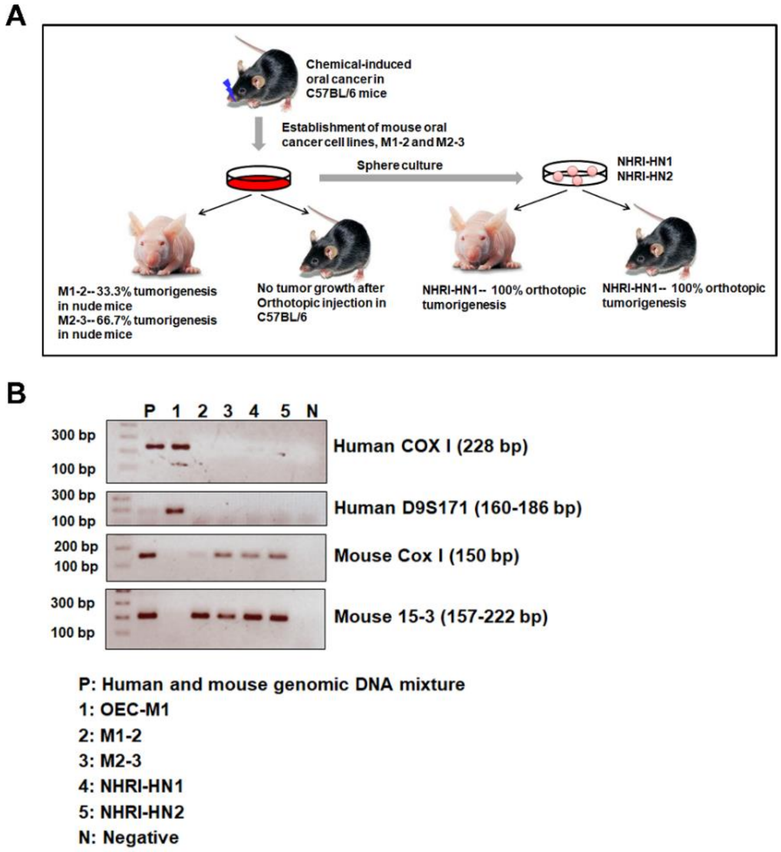

Figure S1. Establishment and genetic identification of mouse oral squamous cell carcinoma (OSCC) cells. (**A**) The scheme for establishment of mouse OSCC cells. Two mouse OSCC cell lines, named M1-2 and M2-3, from mouse tumor specimens induced by 4-NQO and arecoline were successfully established. M1-2 and M2-3 cells developed into tumors with the efficiency of 33% and 67% in immune-deficient mice, respectively. In contrast, M1-2 and M2-3 cells failed to develop any tumor in syngeneic mice. After in vitro selection by sphere culture, two sublines, named HNRI-HN1 and

NHRI-HN2 cells, were derived from M1-2 and M2-3 cells, respectively. Only NHRI-HN1 cells successfully developed into tumors in immunodeficient and immunocompetent mice. (**B**) Inverted images of polymerase chain reaction (PCR) products amplified with human- or mouse-specific primer pairs of COX I gene. Amplification of human D9S171 and mouse 15-3 served as endogenous controls. Amplified fragments were detected by ethidium bromide staining after agarose gel electrophoresis.

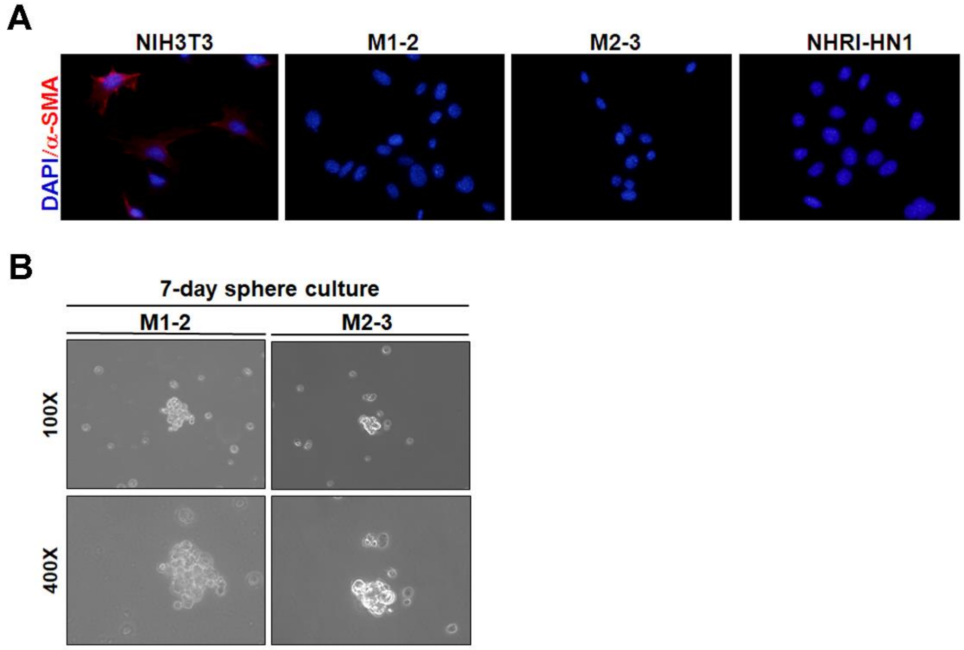

Figure S2. Characterization of mouse OSCC cell lines. (**A**) Representative images of α-smooth muscle actin (α-SMA) and DAPI-stained NIH3T3, M1-2, M2-3, and NHRI-HN1 cells under a fluorescent microscope at 400× magnification. (**B**) The representative pictures of seven-day sphere formation from M1-2 and M2-3 cells under bright-field microscope at 100× and 400× magnifications.

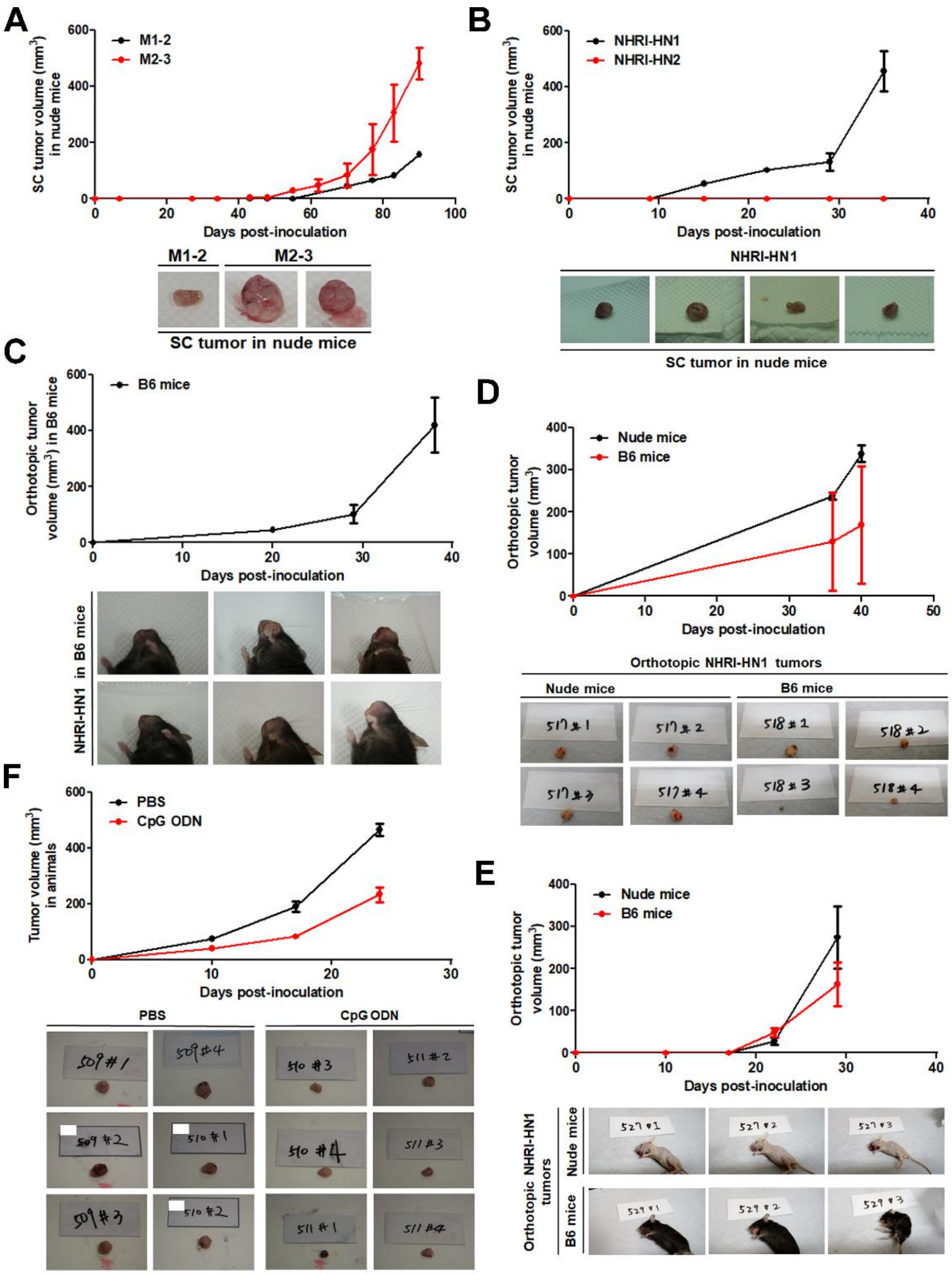

Figure S3. Tumor growth of mouse OSCC cells in nude mice and B6 mice. (**A**) Upper: the tumor growth curves in nude mice subcutaneously injected with 10^6 of M1-2 (n = 1) and M2-3 cells (n = 2). Lower: the developed SC tumors from nude mice sacrificed at 98 days post-inoculation. (**B**) Upper: tumor growth curves in nude mice subcutaneously injected with 10^6 of NHRI-HN1 (n = 4) and NHRI-HN2 cells (n = 4). Lower: the developed SC tumors form nude mice sacrificed at 42 days post-inoculation. (**C**) Upper: the representative tumor growth curve in B6 mice orthotopically injected with 5×10^5 of NHRI-HN1 (n = 6 in the fourth independent experiment). Lower: the tumor bearing mice sacrificed at 38 days post-inoculation. (**D**) Upper: the tumor growth curves in nude mice (n = 4) and B6 mice (n = 4) orthotopically injected with 5×10^5 of NHRI-HN1 cells. Lower: the developed orthotopic tumors from mice sacrificed at 40 days post-inoculation. (**E**) Upper: the tumor growth curves in nude mice (n = 3) and B6 mice (n = 3) orthotopically injected with 5×10^5 of NHRI-HN1 cells. Lower: the tumor bearing mice sacrificed at 36 days post-inoculation. (**F**) Upper: the tumor growth curves in PBS- or CpG-ODN-treated B6 mice orthotopically injected with 2.5×10^5 of NHRI-HN1 cells (n = 6/ group). Lower: the developed orthotopic tumors from PBS- or CpG-ODN-treated mice sacrificed at 29 days post-inoculation.

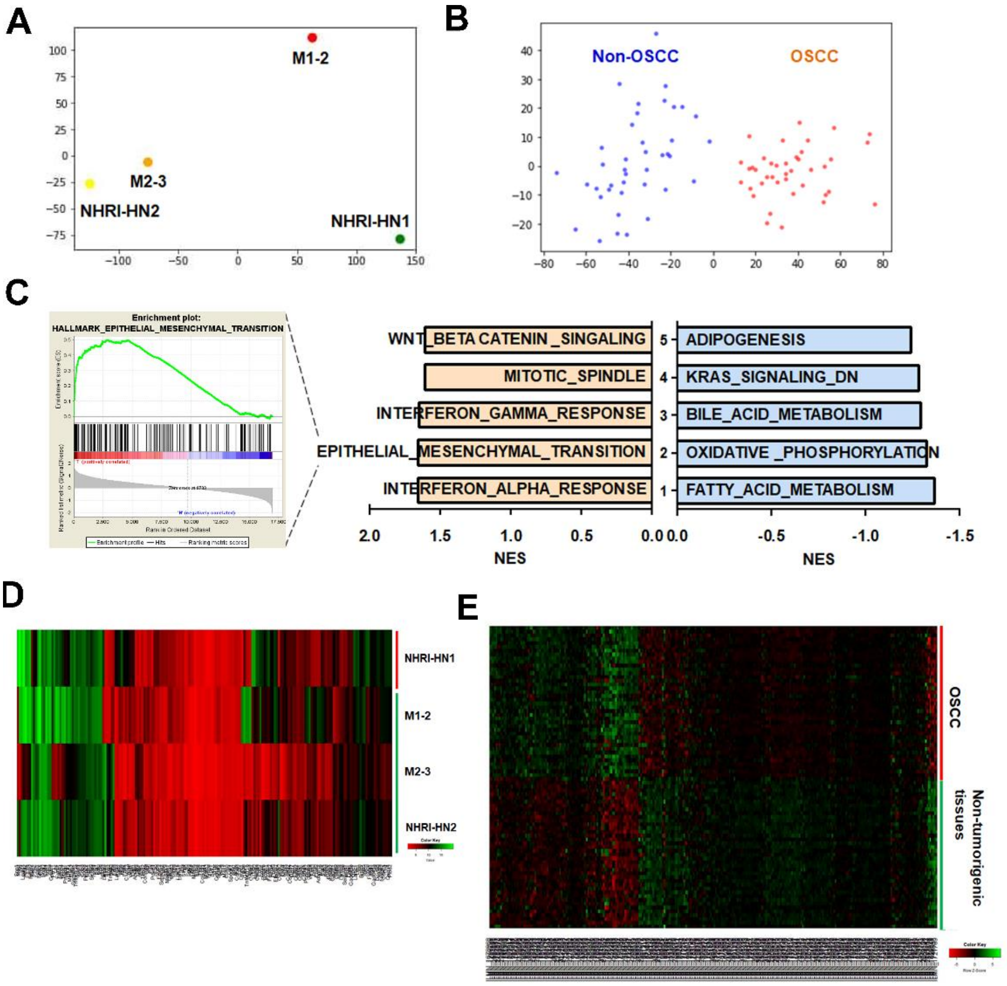

Figure S4. Gene expression analysis of 4 mouse OSCC cell lines and 40 pairs of OSCC and their corresponding adjacent nontumor tissues. (**A**) Clustering analysis using PCA shows that NHRI-HN1 cells was slightly separated from cluster formed by non-tumorigenic M1-2, M2-3 and NHRI-HN2 cells. (**B**) PCA of OSCC tissues and non-tumorigenic tissues shows obviously distinct two clusters. Gene expression profiles were obtained from GSE37991. (**C**) The most enriched hallmark pathways associated with oral tumorigenesis in 40 pairs of human OSCC tissues and adjacent nontumor tissues. Blue is negative value of NES and orange is positive value of NES. (**D**) Heatmap of EMT pathway (184 genes) revealed a significant gene expression pattern between NHRI-HN1 cells and nontumorigenic cells. (**E**) Heatmap of EMT signature (288 genes) showed an obviously different gene expression pattern between OSCC tissues and their adjacent non-tumorigenic tissues. Red color represents up-regulation and green color represents down-regulation.

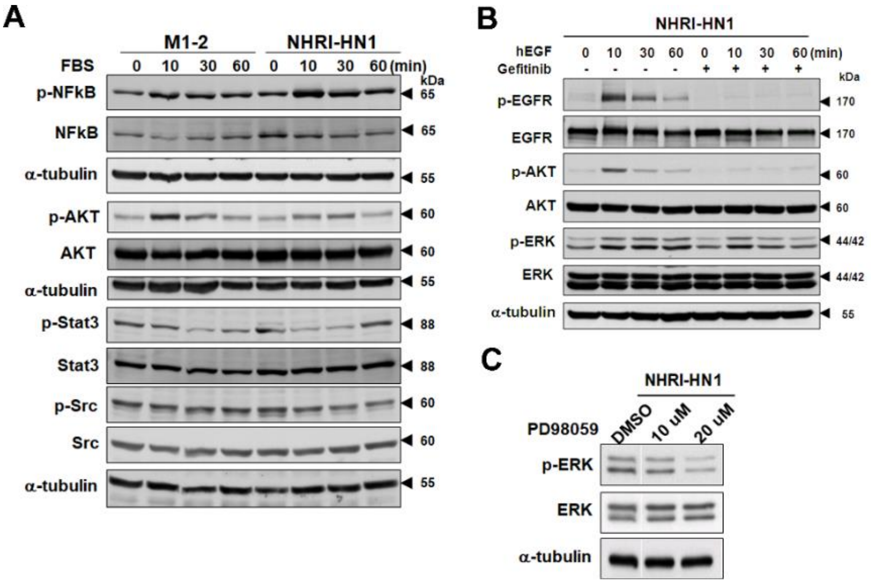

Figure S5. ERK activation in NHRI-HN1 cells. (**A**) Kinase profiling in M1-2 and NHRI-HN1 cells. Immunoblot assays detecting total and phosphorylated NF-kB, AKT, Stat3 and Src in M1-2 and NHRI-HN1 cells upon treatment with FBS for 10, 30, and 60 mins. (**B**) The time course immunoblot assay for total and phosphorylated EGFR, ERK and AKT in hEGF-treated NHRI-HN1 cells, with or without gefitinib at 0, 10, 30, and 60 min. time points. (**C**) ERK activation was inhibited by PD98059 in NHRI-HN1 cells.

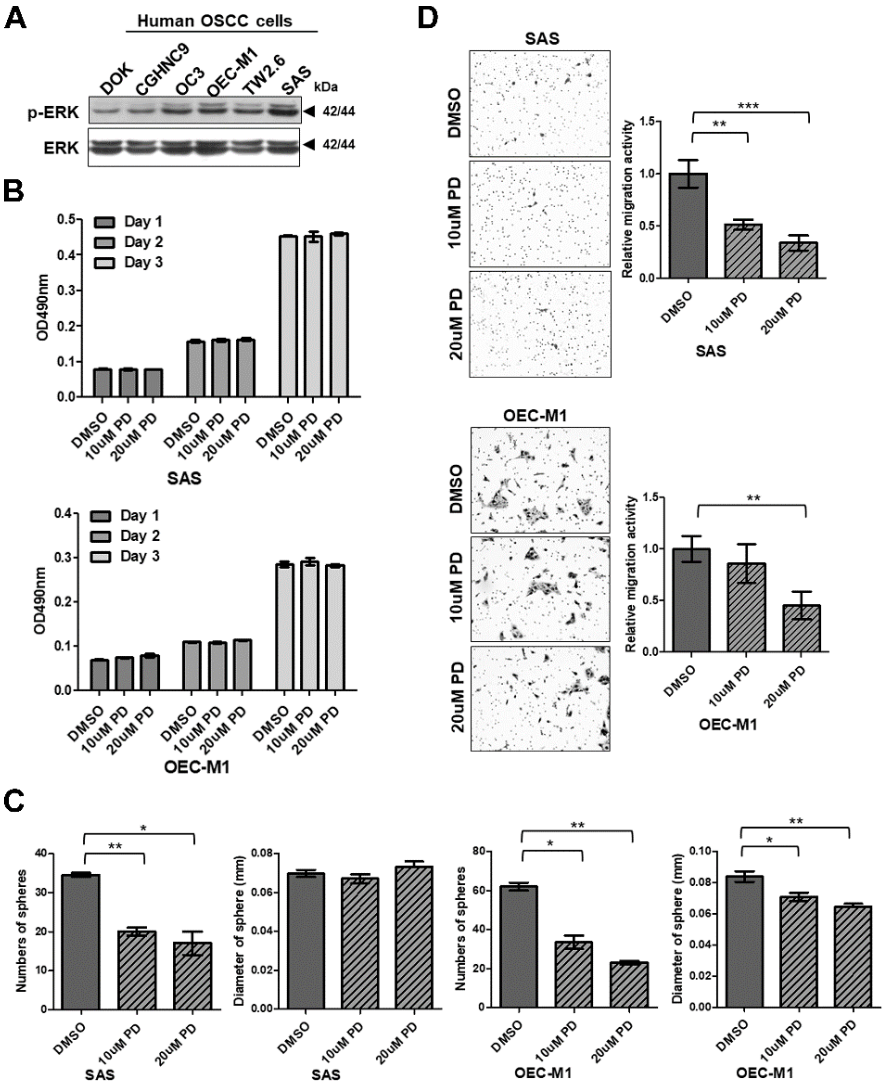

Figure S6. Blockage of ERK activation by PD98059 inhibited cell migration and stemness of human OSCC cells. (**A**) ERK activation in human OSCC cell lines. (**B**) The cell growth in (Upper panel) SAS and (Lower panel) OEC-M1 cells treated with PD98059, analyzed by MTS assay for three days. (**C**) The sphere-forming activities in (Left panel) SAS and (Right panel) OEC-M1 cells treated with PD98059, assessed by sphere culture. The average sphere numbers and diameters of sphere were shown. (**D**) Representative data showing the relative migration activity of (upper panel) SAS and (Lower panel) OEC-M1 cells treated with PD98059. Left panel: migrated cells observed at 100× magnification. Right panel: the relative migration activity was calculated by normalizing the mean of migrated cells per field to that of control cells. Error bars represent SE; * p <0.05; ** $p < 0.01$; *** $p < 0.001$.

Figure 4D

	N-cadherin	α-catenin	Vimentin	E-cadherin	β-catenin	Twist	Snail	Slug
M1-2	1	1	1	1	1	1	1	1
NHRI-HN1	1.12	2.17	2.19	0	1.67	4.84	3.68	1.29

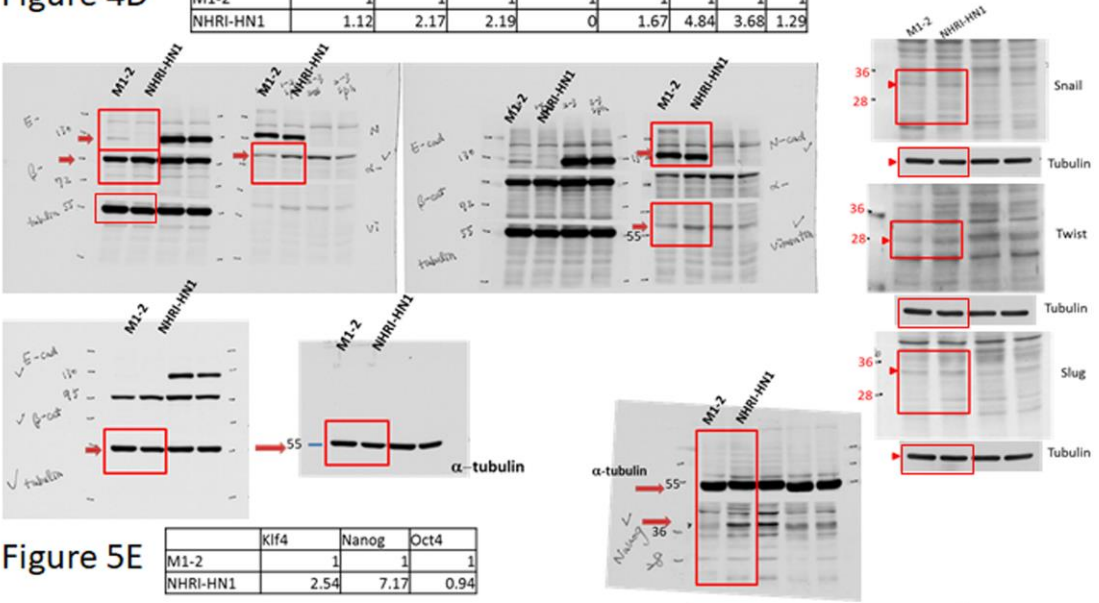

Figure 5E

	Klf4	Nanog	Oct4
M1-2	1	1	1
NHRI-HN1	2.54	7.17	0.94

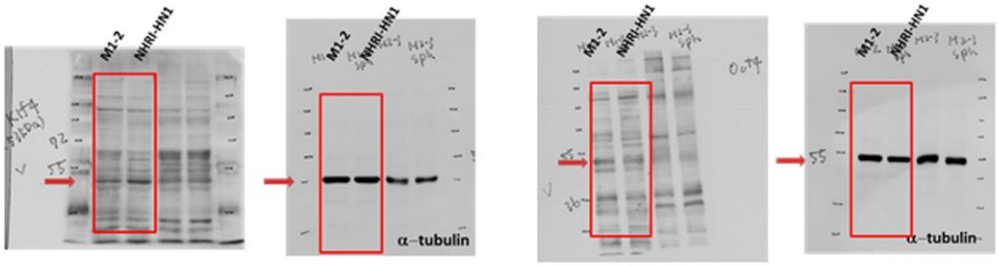

Figure 6A

	p-EGFR	EGFR	p-ERK	ERK
M1-2 0	1.0	1.0	1.0	1.0
M1-2 10	2.2	1.3	2.6	0.8
M1-2 30	1.5	1.3	2.0	0.9
M1-2 60	1.2	1.3	1.0	0.9
NHRI-HN1 0	1.0	1.0	1.0	1.0
NHRI-HN1 10	1.0	0.9	1.7	1.1
NHRI-HN1 30	0.9	0.9	1.6	1.1
NHRI-HN1 60	0.9	0.8	1.2	0.9

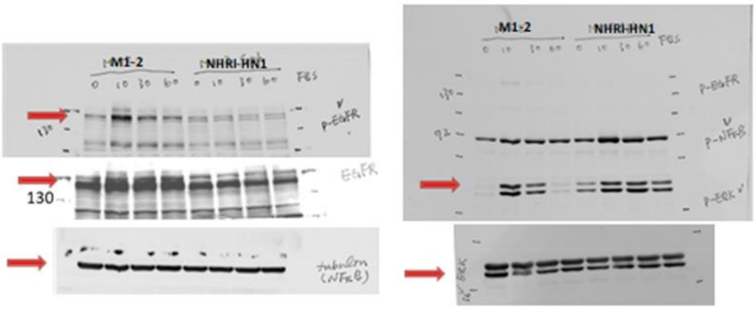

Figure 6G

	Klf4	Nanog	Vimentin	E-cadherin	Snail	N-cadherin	Twist
NHRI-HN1 0	1	1	1	0	1	1	1
NHRI-HN1 10	0.86	0.92	1.31	0	2.2	0.98	0.85
NHRI-HN1 20	1.16	0.71	1.78	0	2.3	0.96	1.04

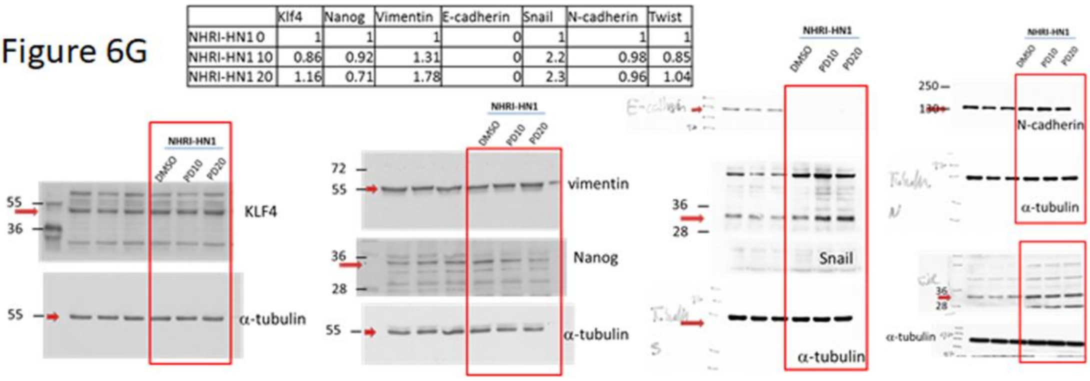

Figure S1B

	hCOXI/ D9S171	mCOXI/ 15-3
Positive	5.76	21.63
OEC-M1	1.00	0.00
M1-2	0.00	1.00
M2-3	0.00	18.64
NHRI-HN1	0.00	9.48
NHRI-HN2	0.00	16.01
Negative	0.00	0.00

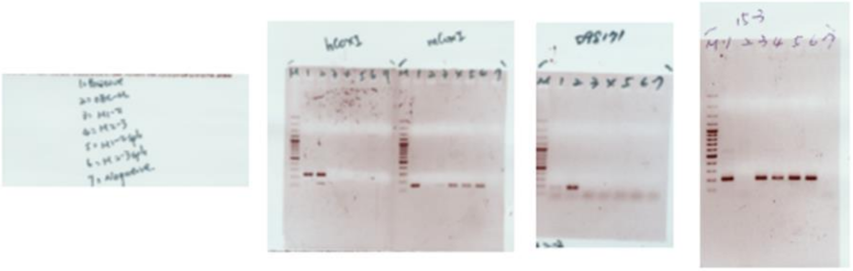

Figure S4A

	p-NFκB	NFκB	p-AKT	AKT	p-Stat3	Stat3	p-Src	Src
M1-2 0	1.0	1.0	1.0	1.0	1.0	1.0	1.0	1.0
M1-2 10	1.2	1.0	2.4	0.8	0.8	0.9	0.9	0.8
M1-2 30	1.2	1.2	1.4	0.8	0.8	1.1	1.1	1.1
M1-2 60	1.1	1.2	1.2	1.0	0.8	0.9	1.0	0.9
NHRI-HN1 0	1.0	1.0	1.0	1.0	1.0	1.0	1.0	1.0
NHRI-HN1 10	1.4	0.9	1.6	1.0	0.6	0.8	0.7	0.8
NHRI-HN1 30	1.2	0.9	1.6	0.9	0.6	0.8	0.7	0.9
NHRI-HN1 60	1.0	0.8	1.1	1.0	0.9	0.9	0.6	0.8

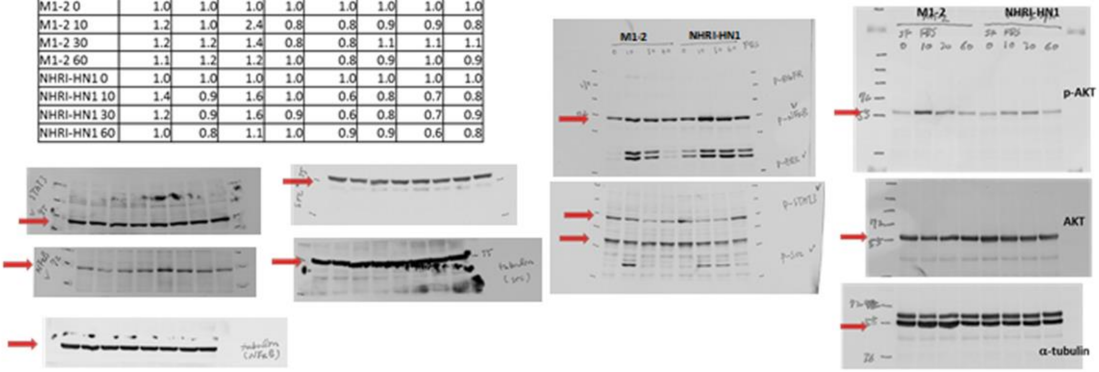

Figure S4B

	p-EGFR	EGFR	p-AKT	AKT	p-ERK	ERK
NHRI-HN1 0 -	1.0	1.0	1.0	1.0	1.0	1.0
NHRI-HN1 10 -	2.7	1.1	1.9	0.9	1.6	1.0
NHRI-HN1 30 -	1.6	0.8	1.1	0.8	1.5	0.9
NHRI-HN1 60 -	0.9	0.6	0.9	0.8	1.3	0.8
NHRI-HN1 0 +	0.8	0.8	0.8	0.8	0.9	0.8
NHRI-HN1 10 +	0.9	0.9	0.9	0.9	1.4	0.9
NHRI-HN1 30 +	0.9	0.7	0.8	0.9	1.0	0.9
NHRI-HN1 60 +	0.9	0.7	0.9	0.9	0.9	0.9

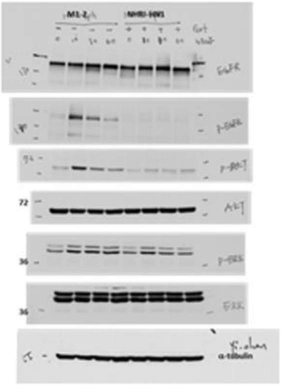

Figure S4C

	p-ERK	ERK
NHRI-HN1 DMSO	1.0	1.0
NHRI-HN2 10 µM	0.9	1.1
NHRI-HN2 20 µM	0.6	1.1

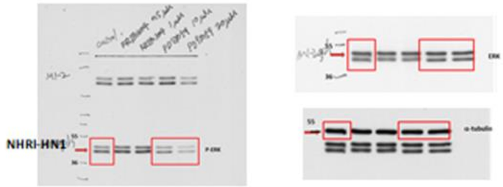

Figure S5A

	p-ERK/ERK
DOK	1
CGHNC9	1.51
OC3	2.06
OEC-M1	1.88
TW2.6	1.93
SAS	2.87

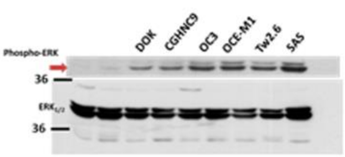

Figure S7. The uncropped scans of western blots from the main and supplementary figures. Protein levels were normalized to an internal control. Ratios were determined by dividing the normalized protein levels in testing cells by that in control cells.

© 2019 by the authors. Submitted for possible open access publication under the terms and conditions of the Creative Commons Attribution (CC BY) license (http://creativecommons.org/licenses/by/4.0/).
